# Partial purification and characterization of glutathione S-transferase from the somatic tissue of *Gastrothylax crumenifer* (Trematoda: Digenea)

**DOI:** 10.14202/vetworld.2017.1493-1500

**Published:** 2014-12-21

**Authors:** Sakil Ahmed, Aamir Sohail, Sabiha Khatoon, Shabnam Khan, Mohammad Khalid Saifullah

**Affiliations:** 1 Section of Parasitology, Department of Zoology, Aligarh Muslim University, Aligarh, Uttar Pradesh, India; 2 Department of Biochemistry, Aligarh Muslim University, Aligarh, Uttar Pradesh, India

**Keywords:** *Bubalus bubalis*, *Gastrothylax crumenifer*, glutathione S-transferase, purification, somatic tissue

## Abstract

**Aim:**

Aim of the present study was to carry out the partial purification and biochemical characterization of glutathione S-transferase (GST) from the somatic tissue of ruminal amphistome parasite, *Gastrothylax crumenifer* (Gc) infecting Indian water buffalo (*Bubalus bubalis*).

**Materials and Methods:**

The crude somatic homogenate of Gc was subjected to progressive ammonium sulfate precipitation followed by size exclusion chromatography in a Sephacryl S 100-HR column. The partially purified GST was assayed spectrophotometrically, and the corresponding enzyme activity was also recorded in polyacrylamide gel. GST isolated from the amphistome parasite was also exposed to variable changes in temperature and the pH gradient of the assay mixture.

**Results:**

The precipitated amphistome GST molecules showed maximum activity in the sixth elution fraction. The GST subunit appeared as a single band in the reducing polyacrylamide gel electrophoresis with an apparent molecular weight of 26 kDa. The GST proteins were found to be fairly stable up to 37°C, beyond this the activity got heavily impaired. Further, the GST obtained showed a pH optima of 7.5.

**Conclusion:**

Present findings showed that GST from Gc could be conveniently purified using gel filtration chromatography. The purified enzyme showed maximum stability and activity at 4°C.

## Introduction

Amphistomes are digenetic trematodes comprising a characteristic posteriorly located muscular acetabulum. There are more than 70 species of amphistome parasites all over the world [1], particularly in the hot and humid land stretches. They are known to parasitize a wide variety of small and large ruminants and are responsible for substantial economic loss. In India, reports on the prevalence of these flukes have been archived from all the major provinces. *Gastrothylax crumenifer* (Gc) is a commonly found amphistome parasite infecting the rumen of the Indian Water buffalo, *Bubalus bubalis* in this part of north India. In general, the adult forms of amphistome parasites manifest diminished pathogenicity, but the immature rumen flukes during their course of migration lead to severe pathological disturbances including hemorrhagic inflammation in the wall of alimentary tract [2]. In India, several outbreaks of acute amphistomosis associated with high mortality among young sheep, goats, cattle, and buffaloes have been recorded [3-6]. In the absence of commercial vaccine, chemotherapy is the only way to deal with these rampant and neglected epidemic infections, but reports on emerging anthelmintic resistance have necessitated the quest to find new drug targets and promising vaccine candidates.

Glutathione S-transferases (GSTs) are a diverse family of multifunctional proteins that find widespread distribution in the aerobic organisms. GSTs mediate the covalent addition of the tripeptide glutathione (GSH) to a structurally diverse set of electrophiles [7]. GST enzymes are involved in the active detoxification of xenobiotics by conjugation to GSH. They are also known to neutralize endogenous secondary metabolite formed during oxidative stress [8]. These enzymes occur in multiple forms and catalyze a multitude of reactions involving electrophilic functional groups [9]. The GST has got a principal role in the process of inactivating a wide range of exogenous/endogenous toxic molecules and to turn them into water-soluble compounds. GSTs gained vital significance in parasites as the main detoxification system due to the lack of cytochrome P450 (CYP450) activity [10]. It has been postulated that GSH mediated antioxidant system is responsible for the prolong survival of helminth parasites in the mammalian definitive host. Occurrence of GSTs in helminth parasites protect them from the reactive oxygen species (ROS) generated during normal metabolism and by the immune effector cells of the invaded host [11,12]. The inherent capacity of helminth GSTs to neutralize cytotoxic components furnished by ROS of host origin on cell membrane strengthens the potential of GSTs as a protective tool against the host immune response. The inhibition of helminth GSTs dismantles the parasite defense against mounting oxidative stress and immunogenic attack of the host [13,14]. These facts place them as targets for the development of vaccines or chemotherapeutic agents [15,16]. The discovery and exploration of biochemical attributes of GSTs in helminth parasites have resulted into elaborate testing of this enzyme form as a target immunoprotective antigen.

There are many reports available on these vaccination experiments [17-20]. Therefore, to cater to that need, preliminary studies were designed and executed to purify and characterize GST from Gc to generate a baseline data which could further be exploited for immune and chemotherapeutic assessments to check amphistome infections in domesticated ruminants.

## Materials and Methods

### Ethical approval

Ethical approval is not necessary to pursue this type of study.

### Collection of parasites

Mature and active Gc amphistome parasites were collected from the rumen of the Indian water buffalo (*B. bubalis*) slaughtered at Aligarh abattoirs. The parasites were thoroughly washed in phosphate buffered saline (100 mM, pH 7.4) and then briefly rinsed in the same washing buffer containing 0.01% penicillin-streptomycin (Merck, Germany) solution to remove any possible microbial contamination.

### Preparation of somatic extracts

The parasites were homogenized in a chilled mortar-pestle over ice in cold phosphate buffer solution (100 mM, pH 7.4). The sample was centrifuged at 10,000×*g* for 10 min in a refrigerated centrifuge (Hitachi, Japan) and the supernatants were collected as soluble protein fractions.

### Protein estimation

The protein contents were estimated following the dye binding method of Bradford [21] as modified by Spector [22]. Bovine serum albumin was used as a standard.

### Ammonium sulfate precipitation

The soluble proteins of somatic extract of Gc were fractionated between 0-20%, 20-40%, 40-60%, 60-80%, and 80-100% ammonium sulfate saturation in a stepwise ascending grade of protein salting out method. After 4 h, the precipitated proteins from each of the fractions were procured by centrifugation at 10,000×*g* for 30 min at 4°C in a cooling centrifuge (Remi, India).

### Dialysis of samples

A small portion of the supernatants from each salting out step was saved and the precipitate was dissolved in a minimum volume of 100 mM sodium phosphate buffer (pH 7.4). The supernatant and the precipitate obtained from each salting out step were dialyzed separately thrice against 1200 ml of the same buffer overnight at 4°C to remove ammonium sulfate content from the protein samples.

### Protein estimation of each fraction

The protein content of the dialyzed supernatant and precipitated samples were estimated as mentioned earlier.

### Assay of GST enzyme

Following protein estimation, all the supernatant and ammonium sulfate precipitated samples of Gc were assayed spectrophotometrically to determine the activity of GST enzyme. The precipitated fractions with maximum GST specific activity were selected and processed for gel filtration chromatography.

### Gel filtration chromatography

A Sephacryl S 100-HR column (Sigma-Aldrich, USA) was prepared as recommended by Peterson and Sober [23] with necessary adjustments and modifications. Pre-swollen gel suspended in ethanol was soaked in sufficient amount of double distilled water and washed at least thrice. The finer resin fragments were removed by suspending the gel in two- to four-fold excess of 100 mM sodium phosphate buffer, pH 7.4 and the gel was allowed to settle down. A glass column (70 cm×2 cm) was mounted on a sturdy vertical support after introducing the glass wool on its opening near the bottom end which was fitted with rubber tubing. Following the clamping of rubber tubing, the column was filled to one-third of its length with operating buffer to check leaks and flush air bubbles from the dead space. The de-aerated gel slurry was poured with the help of a glass rod into the column with care to avoid generating air bubbles. The column was left to stand overnight. Flow rate was increased gradually, and after achieving a constant flow rate (higher than that required for final elution), the column was adjusted to the required flow rate. The packed column was thoroughly washed with two-bed volumes of operating buffer (100 mM sodium phosphate buffer, pH 7.4). To check uniform packing and to determine void volume of the column, 2% (w/v) solution of blue dextran in 100 mM sodium phosphate buffer (pH 7.4) was passed through the column. The volume of the blue dextran and protein solution applied was not more than 2-3% of the total bed volume. The dialyzed sample was subjected to gel filtration chromatography on Sephacryl S-100-HR column equilibrated with 100 mM sodium phosphate buffer, pH 7.4 at 4°C. The flow rate of the column was set at15 ml/h during the process of filtration. Fractions of 5 ml were collected and assayed for protein content and GST activity. Homogeneity of the purification was analyzed by 12% sodium dodecyl sulfate polyacrylamide gel electrophoresis (SDS-PAGE).

### Collection of eluents

The eluents containing protein samples were collected into 14 subsequent fractions (5 ml) and were assayed for protein content and GST activity.

### GST activity in each eluent

The eluted fractions showing detectable amount of protein content were assayed to determine GST enzyme activity in a UV-visible spectrophotometer (Taurus Scientific, U.S.A). GST activity was determined by the method of Habig *et al*. [24] with minor modifications. The assay was performed in a total volume of 3.0 ml reaction mixture containing 300 µl of 1.0 mM reduced GSH, 10 µl of 1.0 mM 1-chloro-2,4-dinitrobenzene (CDNB), and 50 µl of protein samples. The remaining volume was adjusted with 0.1 M sodium phosphate buffer (pH 6.5). CDNB and GSH were dissolved in ethanol and 0.1 M sodium phosphate buffer (pH 6.5), respectively. The control assay mixture did not have any protein (enzyme) sample. The assay was carried out at least in three replicates. The change in absorbance at 340 nm was recorded for 3 min. The change in absorbance was calculated and used for determining the enzyme specific activity. The GST activity is defined as the amount of enzyme that catalyzes the formation of 1.0 µmol of S-(2,4-dinitrophenyl) GSH/min/mg protein under the standard assay conditions. The unit of enzyme activity is expressed as nmoles/mg protein/minute.

### Molecular weight determination of GST enriched fraction by SDS-PAGE

The eluted fractions showing highest activity for GST were subjected to SDS-PAGE to assess the homogeneity of purification as well as relative percentage of GST protein in the GST enriched fractions. All the samples and buffers used in this study were filtered through 0.22 μm Millipore filters to remove small particles and the gel solutions were additionally degassed before gel polymerization.

### Protein loading and electrophoresis

Eluted fractions containing highest GST activity in Gc were separately mixed with the SDS sample buffer containing β-mercaptoethanol in the ratio of 1:2 (Sample: Sample buffer) and then the mixture was heated for 3-5 min at 95°C in water bath. Samples were loaded in different wells along with the standard protein markers (Precision Plus Protein^™^ Dual Color Standard; BIO-RAD) in a separate lane. After that, the gels were placed in a Benchtop Mini-Boat gel electrophoresis assembly and then running buffer was poured into the buffer tank. Electrophoresis [25] was carried out at 100 V for 60 min at RT. The gels were then carefully removed and thoroughly washed twice with distilled water before incubation in CBBR-250 solution overnight at RT. The over stained gels were destained, photographed, and analyzed.

### Native-PAGE for GST activity determination

#### Gel preparation

To resolve the isozyme pattern and activity of GST enzyme molecules, the polyacrylamide gels were cast between glass cassettes in an electrophoresis assembly. A 12% separating gel and stacking gel of 5% concentration were prepared.

#### Protein loading and electrophoresis

Samples (desired eluted fractions of Gc) were mixed with the non-reducing sample buffer in the ratio of 1:2 (sample: sample buffer) and then the mixture was held at 4°C for 2-4 h. The prepared samples were loaded and electrophoresis was done at 100 V for 90 min.

#### Activity of GST in native polyacrylamide gel

The activity of GST enzyme in gel was performed according to the protocol of Ricci *et al*. [26]. The electrophoresed gels were then incubated in a series of reagents to develop achromatic bands depicting GST isozyme.

#### Photography of stained gels

The stained gels were scanned on a computer-driven laser scanner for analysis and all the images were saved for further analysis as well as to maintain digital record in the laboratory.

#### Effect of pH on partially purified GST

Variation in the enzyme activity of partially purified GST obtained from Gc was examined at various pH values (5.0, 5.5, 6.0, 6.5, 7.0, 7.5, 8.0, 8.5, and 9.0). Purified GST enzymes (1 mg) from the amphistome parasites were incubated separately in 1 ml of 100 mM sodium phosphate buffered solution having different pH at a constant temperature of 4°C for 30 min. Following incubation, each sample was assayed for GST activity as mentioned above.

#### Effect of temperature on partially purified GST

The variation in specific activity of GST from Gc was also investigated as a function of five different temperature (4, 25, 37, 60, and 80)°C. Purified GST enzymes (1 mg) were incubated separately in 1 ml of sodium phosphate buffered solution (100 mM, pH 7.4) pre-maintained at specific temperatures, for 30 min. Following incubation, each sample was assayed for GST activity according to the methods mentioned earlier.

## Results

The present work deals with the partial purification of GST protein from the somatic tissues of amphistome parasites, Gc. The methodology involved two steps isolation process using tissue homogenates. The aqueous protein extracts were subjected to ammonium sulfate precipitation (40-60%) for Gc. The precipitated protein fractions from the flukes were passed through a Sephacryl S-100 HR column. Different steps of typical purification process of GST proteins, their specific enzyme activity, fold purification, and percent yield are summarized in Table-1. Fractionation of the soluble proteins with ammonium sulfate decreased the total amount of protein obtained in the subsequent precipitate. The ammonium sulfate precipitation (40-60%) revealed 47.74% yield of the total activity in crude protein but the GST protein showed 2.78-fold purification as compared to the crude homogenate of Gc.

**Table-1:** Different steps involved in the purification of GST proteins obtained from the somatic tissues of *Gastrothylax crumenifer* (GcGST).

Steps of purification	Total volume (ml)	Total protein (mg)	Total activity (n moles)	Specific activity (nmoles/mg protein/min)	Fold purification	Percent (%) yield
Crude homogenate	65	1187.355	1315.300	1.108	1	100
(40-60)% Ammonium sulfate precipitate	10	203.510	628.000	3.086	2.785	47.746
Gel filtration (Sephacryl S-100 HR)	6	9.920	244.800	24.677	22.272	18.612

GST=Glutathione S-transferase

### Gel filtration chromatography

**Figure-1:**
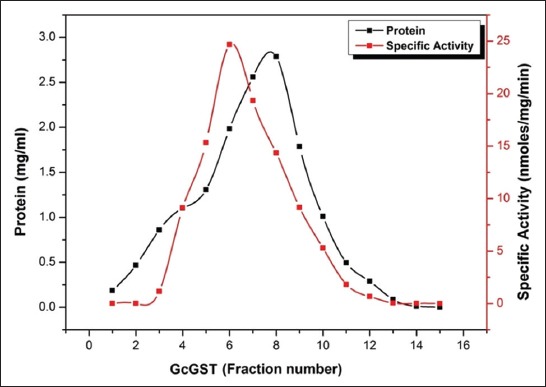
Elution profile of 40-60% ammonium sulfate precipitated protein fraction of *Gastrothylax crumenifer* as obtained by gel filtration chromatography on Sephacryl S-100 HR gel matrix.

The ammonium sulfate precipitated proteins (40-60%) obtained were chromatographed on a Sephacryl S-100 HR column pre-equilibrated with 100 mM sodium phosphate buffer (pH 7.4). Single peak showing highest GST specific enzyme activity (24.677 nmoles/mg protein/min) was obtained for the rumen flukes as shown in Figure-1. The sixth eluted fraction corresponding to the peak was used for further analyses. It was observed that the size exclusion chromatography could purify the GST protein by 22.27-folds from crude homogenates of Gc. The percent yields of GST protein after size exclusion chromatography in Gc were found to be 18.61 as compared to their corresponding crude forms (Table-1).

### Homogeneity of the purified GST proteins

Eluted GST proteins from Gc showed a single peak corresponding to highest enzyme specific activity suggested a homogenous soluble extract preparation (Figure-1). In addition, the preparations had minimum interference with the ions of sodium and phosphates as are evident from the symmetric peaks and patterns of eluting fractions in terms of protein content and GST specific activity. Physical evidence for homogeneity was further provided by PAGE.

### Reducing SDS-PAGE

**Figure-2:**
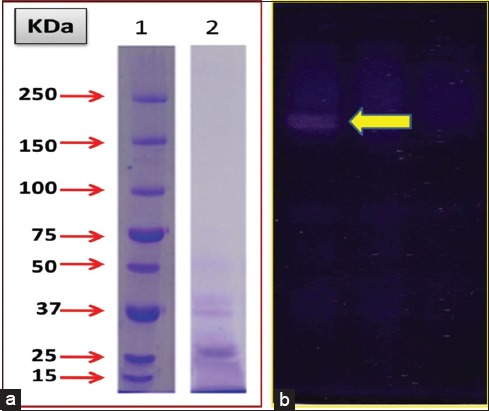
(a) Sodium dodecyl sulfate polyacrylamide gel electrophoresis (PAGE) analysis of purified glutathione S-transferase (GST) from *Gastrothylax crumenifer* (Gc) in a 12% gel. Lane 1 - standard protein marker (SPM), Lane 2 - purified GST from Gc (GcGST). (b) In-gel activity of purified GST from Gc in 10% native PAGE gel. The activity band appears as an achromatic zone on a blue insoluble formazan background.

Partially purified GST proteins from Gc (Eluted fraction-6) were analyzed on PAGE under reducing and non-reducing (in absence of SDS and β-mercaptoethanol) conditions. The PAGE (12%) profile under reducing ­conditions showed that the GST migrated as a single prominent band in the parasites under study (Figure-2a). The specific polypeptides corresponding to GST monomeric proteins in the rumen amphistomes were observed to be lying in the low molecular weight range.

### Non-reducing (native) PAGE

Partially purified GST proteins from the eluted fraction number 6 for Gc (GcGST) were analyzed on native PAGE under non-reducing conditions. Subsequent staining of the gel matrix for in-gel activity of GST revealed a single achromatic band over a blue insoluble formazan dark background (Figure-2b).

### Properties of the partially purified GST proteins

#### Molecular weight determination

Analysis of the resolved polypeptide on a 12% SDS polyacrylamide gel in a Bio Rad Gel Documentation system revealed a single band of 26 kDa polypeptide corresponding to GST monomer (Figure-2a).

#### Effect of pH on partially purified GST

**Figure-3:**
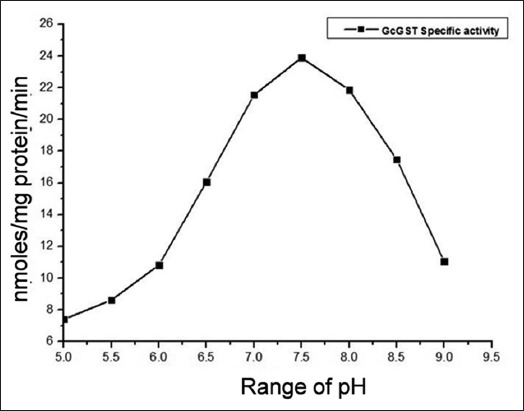
The pH stability of the purified glutathione S-transferase (GST) proteins from *Gastrothylax crumenifer* in 100 mM sodium phosphate buffer having different pH (5.0-9.0). Purified GSTs (1 mg) were incubated in 1 ml of phosphate buffer solution at 4°C for 30 min.

Effect of pH on the specific activity of partially purified GST was examined at various pH values ranging from 5.0 to 9.0 (Figure-3). The GST activities in the parasitic flukes were seen to be drastically affected at both the extreme of the test range. The GST proteins were found to have considerable enzymatic activities in a pH range of 6.5-8.5 in the parasite under study. Maximum stability of the catalytic properties of GST proteins was observed at pH 7.5 (Figure-3). A rise in pH from 7.5 to 8.0 resulted in an evident decrease in the enzyme activity. This suggests that the GST enzyme in this amphistome parasite has specific pH optima.

#### Effect of temperature on partially purified GST

**Figure-4:**
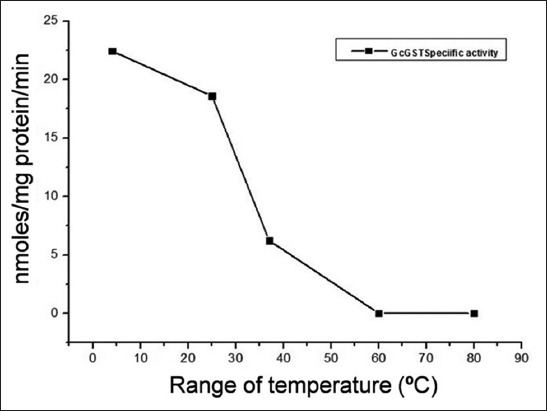
Thermal stability of the purified glutathione S-transferase (GST) proteins from *Gastrothylax crumenifer* in 100 mM sodium phosphate buffer (pH 7.4) pre-maintained at five different temperatures (4, 25, 37, 60, and 80°C). Purified GSTs (1 mg) were incubated in 1 ml of phosphate buffer solution having different temperatures for 30 min.

Alteration in the specific activity of GST from Gc was also investigated as a function of temperature in a range from 4 to 80°C for 30 min. The GST protein remained considerably active within the temperature of 4-37°C for the parasite species (Figure-4). However, a rise in temperature from 25 to 30°C resulted into sharp decline in the GST specific activity profile in the parasites under study (Figure-4). When the temperature was raised from 4 to 25°C, the abrupt slump in the activities of GST proteins was recorded. This suggested that GST from Gc is appreciably robust in terms of minor thermal fluctuations at lower temperature ranges.

## Discussion

GSTs exist in all living organisms and they are involved in various detoxification pathways and antioxidant processes. Since, helminth parasites lack p-450 monoxygenases, the Phase-II biotransformations are carried out by GST isoforms and hence are of cardinal importance [27]. GSTs perform several important functions in the body and are associated with several pathological conditions including cancer [28], rheumatoid arthritis [29], osteoporosis [30], renal dysfunctions [31], cardiovascular diseases [32,33], and Alzheimer’s disease [34]. GSTs have been purified and characterized from various helminth parasites including nematodes, trematodes, and cestodes such as *Clonorchis sinensis* [35], *Setaria digitata* [36], *Schistosoma mansoni* [37], *Schistosoma japonicum* [38], *Fasciola hepatica* [39], *Necator americanus* [40], and *Echinococcus granulosus* [41]. Due to lack of information on GSTs from a neglected group of digenetic trematodes, the amphistome parasites, present work has been carried out. To the best of our knowledge, this is the first description about the purification and properties of GST enzymes obtained from the amphistome parasites, Gc. It was envisaged that a thorough study on GST from the ruminal amphistomes will shed some light toward better understanding of GSTs of helminth origin. This will be useful in generating a base line data so that the salient enzymatic properties can be compared with other known GST from different parasitic fauna. In the present work, GST was purified from Gc by the method as described in the materials and method section. The two step procedure involved ammonium sulfate fractionation and gel filtration chromatography. The simple isolation procedure provided a percent yield of 18.61- and 22.27-fold purification for Gc. Purification of the GST isozymes from a variety of helminth parasites has been reported using a combination of several methods including affinity chromatography, chromatofocusing, gel filtration, and ion exchange chromatography [12,14,42-44]. However, the procedure used in this study is simple and cost-effective which give appreciable yield and fold purification as compared to the values reported in literature for other helminth parasites [36]. The partially purified GST obtained from the amphistome parasites was found to be homogenous on the basis of charge as shown by native-PAGE. In SDS-PAGE under reducing conditions, GST from Gc gave a single band which could be of dimeric nature (native state) with two identical subunits.

GSTs obtained from amphistome parasites were partially purified on a Sephacryl S 100-HR resin matrix. The molecular mass of the subunits comprising the native GST molecule was found to be made up of two equal sub-units of 26 kDa in Gc following denaturing PAGE technique in the presence of β-mercaptoethanol. Subunits of GST with comparable molecular weights (26 kDa) have also been reported in a wide variety of helminths such as *S. mansoni* [37], *S. japonicum* [45], *F. hepatica* [46], *Taenia solium* [42], and *Setaria cervi* [43]. In general, cytosolic GSTs have monomers of 23-28 kDa with an average of 220 amino acids in their sequences. They all share the same tertiary and quaternary dimeric structural features. The dimer may have identical subunits (homodimer) or different subunits (heterodimer) of the same class [47]. In the present study, appreciable stability of the partially purified GSTs from Gc was observed in the broad range of pH (6.5-8.5) as well as temperature (4-37°C). Similar kinds of observations were made with GST purified from *S. digitata* which showed good enzyme activity between 0°C and 40°C, while a sharp decline in activity was observed beyond 40°C with complete loss of activity at 80°C [36]. A similar trend in the GST enzyme activity was reported earlier with temperature optima of around 40°C [42,43] and loss of activity at higher temperatures. The enzyme activity of GSTs from Gc was seen to vary with change in pH. The optimum GST activities for this amphistome were observed at pH 7.5; a similar finding was also reported in GST purified from *S. digitata* [36].

In most of the helminth parasites, CYP450 mediated biotransformation has not been reported. Hence, the GST enzyme system plays an indispensible role in carrying out the Phase II detoxification pathways. Due to this, purified GSTs from various helminth parasites have been screened for immune and chemotherapeutic leads and targets to design and develop a better and reliable parasite control measure. The ­present study was an attempt to purify GST enzyme from an amphistome parasite to determine and obtain the basic biochemical attributes which could help in the formulation of new drug to control the amphistomosis in our farm animals.

## Conclusion

The present study is an attempt to purify and conduct basic biochemical characterization of GST from the rumen infecting amphistome Gc using size exclusion chromatography.

## Authors’ Contributions

SA and AS prepared the study design and carried out the research under the supervision of MKS. SK and SHK isolated the parasites and analyzed the data. The manuscript was drafted and revised by SA and SK under the guidance of MKS. All authors read and approved the final manuscript.
